# Molecular Dynamics Simulation of Kir6.2 Variants Reveals Potential Association with Diabetes Mellitus

**DOI:** 10.3390/molecules29081904

**Published:** 2024-04-22

**Authors:** Mohamed E. Elangeeb, Imadeldin Elfaki, Ali M. S. Eleragi, Elsadig Mohamed Ahmed, Rashid Mir, Salem M. Alzahrani, Ruqaiah I. Bedaiwi, Zeyad M. Alharbi, Mohammad Muzaffar Mir, Mohammad Rehan Ajmal, Faris Jamal Tayeb, Jameel Barnawi

**Affiliations:** 1 Department of Basic Medical Sciences, College of Applied Medical Sciences, University of Bisha, Bisha 61922, Saudi Arabia; 2 Department of Biochemistry, Faculty of Science, University of Tabuk, Tabuk 71491, Saudi Arabia; smalzahrani@ut.edu.sa (S.M.A.); mkhan@ut.edu.sa (M.R.A.); 3 Department of Microbiology, College of Medicine, University of Bisha, Bisha 61922, Saudi Arabia; ameleragi@ub.edu.sa; 4 Department of Medical Laboratory Sciences, College of Applied Medical Sciences, University of Bisha, Bisha 61922, Saudi Arabia; elsadigmohamed69@gmail.com; 5 Department of Clinical Chemistry, Faculty of Medical Laboratory Sciences, University of El Imam El Mahdi, Kosti 27711, Sudan; 6 Department of Medical Laboratory Technology, Prince Fahad Bin Sultan Chair for Biomedical Research, Faculty of Applied Medical Sciences, University of Tabuk, Tabuk 71491, Saudi Arabia; rashid@ut.edu.sa (R.M.); rbedaiwi@ut.edu.sa (R.I.B.); z.alharbi@ut.edu.sa (Z.M.A.); f.tayeb@ut.edu.sa (F.J.T.); jbarnawi@ut.edu.sa (J.B.); 7 Department of Clinical Biochemistry, College of Medicine, University of Bisha, Bisha 61922, Saudi Arabia; mmmir@ub.edu.sa

**Keywords:** nsSNPs, *KCNJ11* gene, diabetes mellitus, bioinformatics, ATP-sensitive potassium channels (K_ATP_ channels), genetic testing

## Abstract

Diabetes mellitus (DM) represents a problem for the healthcare system worldwide. DM has very serious complications such as blindness, kidney failure, and cardiovascular disease. In addition to the very bad socioeconomic impacts, it influences patients and their families and communities. The global costs of DM and its complications are huge and expected to rise by the year 2030. DM is caused by genetic and environmental risk factors. Genetic testing will aid in early diagnosis and identification of susceptible individuals or populations using ATP-sensitive potassium (K_ATP_) channels present in different tissues such as the pancreas, myocardium, myocytes, and nervous tissues. The channels respond to different concentrations of blood sugar, stimulation by hormones, or ischemic conditions. In pancreatic cells, they regulate the secretion of insulin and glucagon. Mutations in the *KCNJ11* gene that encodes the Kir6.2 protein (a major constituent of K_ATP_ channels) were reported to be associated with Type 2 DM, neonatal diabetes mellitus (NDM), and maturity-onset diabetes of the young (MODY). Kir6.2 harbors binding sites for ATP and phosphatidylinositol 4,5-diphosphate (PIP2). The ATP inhibits the K_ATP_ channel, while the (PIP2) activates it. A Kir6.2 mutation at tyrosine330 (Y330) was demonstrated to reduce ATP inhibition and predisposes to NDM. In this study, we examined the effect of mutations on the Kir6.2 structure using bioinformatics tools and molecular dynamic simulations (SIFT, PolyPhen, SNAP2, PANTHER, PhD&SNP, SNP&Go, I-Mutant, MuPro, MutPred, ConSurf, HOPE, and GROMACS). Our results indicated that M199R, R201H, R206H, and Y330H mutations influence Kir6.2 structure and function and therefore may cause DM. We conclude that MD simulations are useful techniques to predict the effects of mutations on protein structure. In addition, the M199R, R201H, R206H, and Y330H variant in the Kir6.2 protein may be associated with DM. These results require further verification in protein–protein interactions, Kir6.2 function, and case-control studies.

## 1. Introduction

Diabetes mellitus (DM) is a long-term condition of glucose intolerance characterized by increased blood sugar. The prevalence of DM is increasing worldwide [1]. DM has very serious impacts due to its complications in the patients, impacting them and their families, societies, and communities all over the world [1]. Types of DM are as follows: Type 1 DM, which is caused by an insulin deficiency due to the destruction of pancreatic beta cells medicated by T-lymphocytes [2]. Type 2 DM results from impaired insulin action and beta cells demise [3]. The third type is gestational DM; it is due to the failure of pancreatic beta cells with a background of impaired insulin action during the gestation period [4]. Other types of DM include maturity-onset diabetes of the young (MODY), which is a group of disorders resulting from heterozygous molecular defects in different genes involved in the development of the pancreas [5]. MODY is caused by defective steps in the secretion of insulin, e.g., pancreatic beta cells sensing and the metabolism of glucose, or K_ATP_ channel activation [6]. Furthermore, neonatal diabetes mellitus (NDM) is caused by genetic defects (e.g., ATP-sensitive K+ channel mutations [7]) and results in reduced beta cells mass or beta cell dysfunction [2]. In addition, NDM, which is persistent, increased blood sugar levels, occurs within 2-6 months in a newborn, and it is a monogenic disorder due to a mutation influencing the development of pancreases or insulin secretion [7]. 

The *KCNJ11* gene, a member of the potassium channel gene family, is found in 11p15.1 with no intron present [8]. The *KCNJ11* gene encodes for the protein Kir6.2, the inward-rectifier potassium ion channel. Kir6.2 and the protein of high-affinity, sulfonylurea receptor 1 (SUR1), form the K_ATP_ channels. The Kir6.2 protein is composed of 390 amino acid residues and has M1 and M2 transmembrane domains and an N-terminus and C-terminus that are localized inside the cell [8]. In pancreatic beta cells, the K_ATP_ channels regulate the secretion and production of insulin via glucose metabolism [8]. In addition, they regulate other physiological processes such as cardiovascular homeostasis and excitation of neurons by responding to the transmitters and status of metabolism [9]. 

The Kir6.2 protein is a major subunit of the K_ATP_ channel [10]. The K_ATP_ channel system is a hetero-octameric complex with a pore; this pore consists of four subunits of Kir6.2 via which potassium ions are transported [11]. The Kir6.2 subunits are associated with four subunits of the larger regulatory subunit –SUR1 [12,13]. All eight protein units (four Kir6.2 and four SUR1) are required to form a functional ATP-sensitive potassium (K_ATP_) channel. This channel is strongly selective for external potassium ions [14]. The potassium flux via the channel is regulated by conformational changes in the region of the helix bundle, G-loop, and filter for selectivity [15]. Gating of the K_ATP_ channel is regulated by different ligands, for example, ATP inhibits Kir6.2 and closes the channel, while the signaling lipid, phosphatidyl-inositol 4,5-bisphosphate (PIP2), activates Kir6.2 and opens the channel [15,16,17,18]. The Kir6.2 subunits contain a conserved and important, disordered N-terminal tail; the disordered tail is a major factor in regulating the state of K_ATP_ and influencing the release of insulin from the pancreatic beta cells [12]. The C-terminal domain of Kir6.2 has roles in K_ATP_ channel trafficking and membrane metabolic regulation [19]. The pancreatic K_ATP_ structures were described at an open pore at 3.1- to 4.0-Å resolution with single-particle cryo-electron microscopy [20]. The opening of the pore is associated with changes in the structure of the Kir6.2 protein (gate of the channel) and the ATP-binding site [20]. There are also conformational changes in the SUR protein, leading to decreased contact surfaces between SUR and Kir6.2 proteins [20]. The pancreatic K_ATP_ channels are different from other inward-rectifier channels in the property of PIP2-independent opening [20], which is associated with a docked cytoplasmic domain when PIP2 is not present [20].

K_ATP_ channels are expressed ubiquitously in the plasma membranes of many tissues such as the myocardium, myocytes, pancreatic beta cells, renal, pituitary, placenta, and central nervous system [21,22]. Bründl et al. conducted MD simulations to investigate the dynamics of amino acid residues associated with gating in Kir6.2 [15]. They report that the Kir6.2 mutation L164P changes the geometry and stability of the K_ATP_ channel and is implicated in NDM [15]. In addition, using MD simulations, the important role of the Kir6.2 N-terminal fragment and the SUR1 protein L0-loop has been shown in the signal transfer that stimulates the release of insulin [23]. Genetic mutations in the genes that encode K_ATP_ channel subunits can result in metabolic and neuronal diseases [24]. Furthermore, gene polymorphisms and mutations in the *KCNJ11* gene were linked with different DM types in previous studies [8,25,26,27,28]. In the current study, we have used bioinformatics tools to study the mutations that potentially influence the structure and function of the Kir6.2 protein and the formation of the K_ATP_ channels. 

## 2. Results

**Figure 1:**
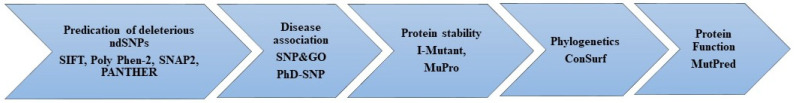
Flow chart of mutational analysis of the *KCNJ11* gene. Analysis of *KCNJ11* gene mutations.

Our analysis (Figure 1) revealed that several nsSNPs in the *KCNJ11* gene might lead to structural alterations in the Kir6.2 protein, potentially affecting its function and interaction with other proteins involved in insulin signaling pathways. Furthermore, molecular dynamics simulations indicated that certain nsSNPs might disrupt the stability and conformational dynamics of the Kir6.2 protein, which could impair its ability to regulate insulin secretion. 

### 2.1. Assessment of the Deleterious Impact of nsSNPs on the KCNJ11 Gene Encodes the Kir6.2 Protein

The functionality of the Kir6.2 protein, encoded by the *KCNJ11* gene, can be influenced by non-synonymous single nucleotide polymorphisms (nsSNPs). Table 1 presents a summary of the outcomes obtained from the utilization of four distinct web servers, namely SIFT, PolyPhen2, SNAP2, and PANTHER. These servers were employed to assess the potential impact of various single nucleotide polymorphisms (SNPs) on the Kir6.2 protein encoded by the *KCNJ11* gene. The table provided displays data regarding the SIFT score predictions for individual amino acid substitutions, which are employed in evaluating the impact on protein functionality. Given their SIFT score of less than 0.05, the data in Table 1 suggest that a large majority of substitutions are likely to have negative effects. This suggests a substantial likelihood of affecting the functionality of proteins. However, it is expected that certain substitutions will have little to no negative effects or very mildly adverse effects, as indicated by their raised SIFT scores. The SIFT score is widely regarded as a good technique for predicting the potential influence of single nucleotide polymorphisms (SNPs) on protein functionality. Table 1 displays the evaluation of the substitution’s impact based on the PolyPhen and SNAP2 scores. These scores are derived from the computational algorithms employed by the respective programs. The PolyPhen score is used to forecast the potential consequences of a substitution, categorizing them as either “probably damaging”, “possibly damaging”, or “benign”. The SNAP2 score is used to forecast the impact of a substitute, categorizing it as either having an effect or having no effect. In order to enhance the reliability of predicting the nature of single nucleotide polymorphisms (SNPs), a combination of SIFT, Poly Phen-2, SNAP2, and PANTHER servers was utilized simultaneously. A total of 440 missense non-synonymous single nucleotide polymorphisms (nsSNPs) were identified by SIFT and PolyPhen2. Upon doing an analysis of the databases with which it is integrated, the system assigns an index score to each reported single nucleotide polymorphism (SNP). A total of 225 nsSNPs, accounting for 51% of the dataset, were identified as potentially deleterious based on SIFT indexing, which assigned them a score of ≤0.05. Furthermore, a total of 166 nsSNPs out of the 225 examined were identified as having a high level of deleteriousness, as indicated by a confidence level of 0.00. A total of 167 nsSNPs were categorized as “probably damaging” according to the PolyPhen indexing system, which relies on structural data and multiple sequence alignment. Additionally, 25 nsSNPs were classified as “possibly damaging”, while 45 were described as benign. By integrating data from SIFT and PolyPhen, a total of 203 non-synonymous single nucleotide polymorphisms (nsSNPs) were eliminated to enhance the precision of the outcomes. Subsequently, 237 non-synonymous single nucleotide polymorphisms (nsSNPs) were submitted to the SNAP2 and PANTHER systems for further validation. The results indicate that a total of 43 nsSNPs have been identified as having a functional impact on the protein. Table 1 presents a review of the findings pertaining to the functional impact of non-synonymous single nucleotide polymorphisms (nsSNPs).

### 2.2. Characterizing Disease-Associated nsSNPs

We utilized a combination of SNP&GO and PhD-SNP tools to identify disease-associated SNPs and assess their impact on the functionality of the Kir6.2 protein encoded by *KCNJ11*. These *in silico* methods allowed us to explore the potential connection between nsSNPs in *KCNJ11* and the development of T2D, shedding light on the functional consequences of these genetic variations. By leveraging the predictive capabilities of these tools, we aimed to gain insights into the role of nsSNPs in the pathogenesis of T2D and their influence on the encoded protein’s function. We utilized the SNP&GO and PhD-SNP tools to detect disease-associated SNPs and forecast the relationship between nsSNPs in the *KCNJ11* gene and T2D, examining their influence on the encoded protein’s function and T2D progression. After applying filtration and reduction techniques, we assessed a restricted set of polymorphisms for their disease association. Subsequently, 43 nsSNPs were subjected to SNP&GO and PhD-SNP analyses to assign a numerical classification indicating their benign or pathogenic nature. An nsSNP is categorized as neutral if its prediction score is below 0.5, while a score exceeding 0.5 indicates disease causality. Among the 43 nsSNPs evaluated, 19 variants were classified as neutral, while the remaining 24 nsSNPs were linked to disease causation. The outcomes of this comprehensive analysis are presented in Table 2.

### 2.3. The Prediction of Protein Stability Consequences

We utilized the I-Mutant and MuPro servers to investigate the potential structural alterations associated with 24 non-synonymous single nucleotide polymorphisms (nsSNPs) in the *KCNJ11* gene. Our findings suggest that 10 of these nsSNPs may exert a destabilizing effect on proteins, potentially impeding their functionality. Additionally, according to the Mut-pro website, structural alterations resulting from eight out of the 24 nsSNPs were found to enhance the functionality of the encoded protein. The results of our analysis are presented in Table 3, providing a comprehensive overview of the predicted effects of these nsSNPs on protein structure and function.

### 2.4. Predicting Pathogenicity and Functional Outcomes of the KCNJ11 Gene nsSNPs

The Mut-pred web server was utilized to predict the pathogenicity and consequential functional alterations resulting from nsSNPs within the Kir6.2 protein. This approach enhances our understanding of how these genetic variants may affect protein function and their association with diseases like T2D through insulin resistance. Mut-pred provides valuable insights into the molecular mechanisms linking nsSNPs in Kir6.2 to disease susceptibility, offering a foundational basis for future investigations. The server’s outcomes aid in predicting the potential impact of missense mutations on protein function, especially in terms of disease association. Furthermore, Mut-pred furnishes comprehensive details about the specific protein attributes likely to be influenced by mutations, encompassing solvent accessibility, secondary structure, and sequence conservation. The results from the Mut-pred analysis (Table 4) indicated that all seven nsSNPs significantly affected protein functionality. Notably, these seven nsSNPs exclusively elicited detrimental impacts on the Kir6.2 protein due to modifications in its structure and intrinsic properties. However, the computational approach suggested that one nsSNP did not manifest any perceptible influence on the protein.

### 2.5. Analysis of Sequence Conservation Patterns

**Figure 2:**
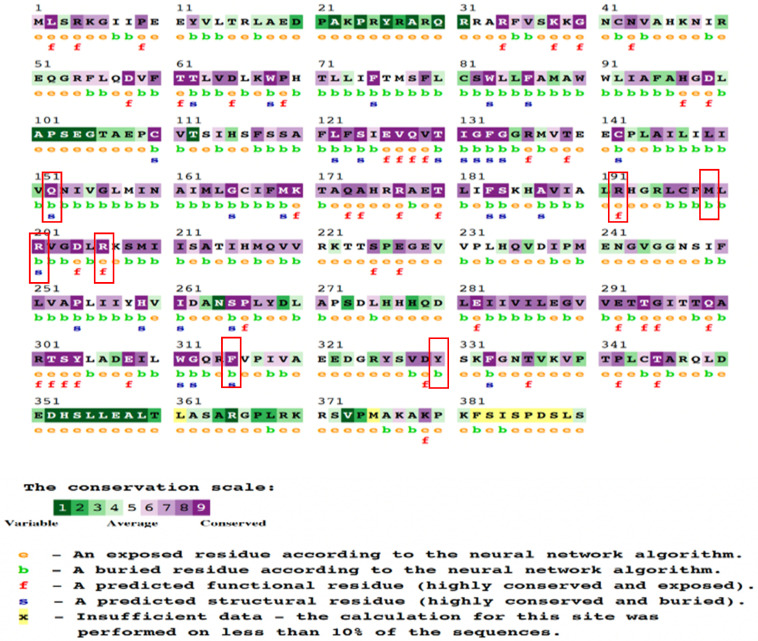
The examination of conserved amino residues in the Kir6.2 structure, focusing on the four mutant variants (M199R, R201H, R206C, Y330H). The ConSurf analysis provided insights into residue conservation. The results are presented with a color spectrum indicating varying levels of confidence in sequence conservation. Within this color spectrum, variable residues are represented by sky-blue, while highly conserved residues are denoted by dark purple.

The ConSurf server was utilized to assess the conservation status of eight nsSNPs associated with T2D. According to the data, this subset of eight nsSNPs demonstrated significant conservation. The analysis was centered on a collection of conserved nsSNPs, of which four exhibited structural and spatial conservation while being located in buried regions. Additionally, two nsSNPs were predicted to be functionally conserved and exposed, whereas two others were identified to be conserved and positioned within inaccessible or buried regions. Employing chromatic schemes, the ConSurf program visually represented sequence conservation levels and provided numerical predictions for modifications on a scale from 1 to 9. A comprehensive overview of the eight nsSNPs is presented in Table 5 and Figure 2, encompassing information on their structural and functional consequences as well as phylogenetic conservation scores. The insights yielded by the ConSurf analysis illuminated the impact of the *KCNJ11* gene’s nsSNPs on DM. By employing this methodology, we gained valuable insights into the evolutionary preservation of amino acid positions affected by these polymorphisms, thus elucidating the potential role of nsSNPs in protein structure. The ConSurf study contributes crucial insights into the molecular mechanisms that underscore the intricate interaction between *KCNJ11* nsSNPs and DM, providing deeper comprehension of disease mechanisms and broader implications for our field’s knowledge.

### 2.6. Predict and Analyze the Structural and Functional Properties of Kir6.2 Using the HOPE Server

The HOPE server presented a 3D structural analysis method to evaluate the impact of specific amino acid mutations on protein function. The M199R mutation resulted in altered size, charge, and hydrophobicity of Kir6.2, in addition to disruption of Kir6.2 folding due to a charge change in a buried residue, while the R201H mutation showed structural changes, domain interactions, and potential effects on Kir6.2 function. The analysis of Y330H led to disrupted hydrogen bonds (HBs), multimer contacts, and core structure alterations potentially affecting Kir6.2 function. Additionally, R206C disrupted the HBs, salt bridges, and domain interactions with potential Kir6.2 functional effects. 

### 2.7. MD Simulation of Kir6.2 Protein and Its Mutants 

Utilizing MD simulations, we gained valuable insights into the dynamic behavior of the protein, its interactions, and potential conformational changes that could be linked to DM. These insights greatly enhance our comprehension of the complex molecular mechanisms underlying this metabolic disorder. Moreover, these results emphasize the significance of accounting for the influence of nsSNPs on protein structure and function in the context of DM. The simulations offer a comprehensive understanding of the protein’s behavior and its potential implications in insulin resistance and metabolic disorders.

#### 2.7.1. Temperature, Pressure, and Density

**Figure 3:**
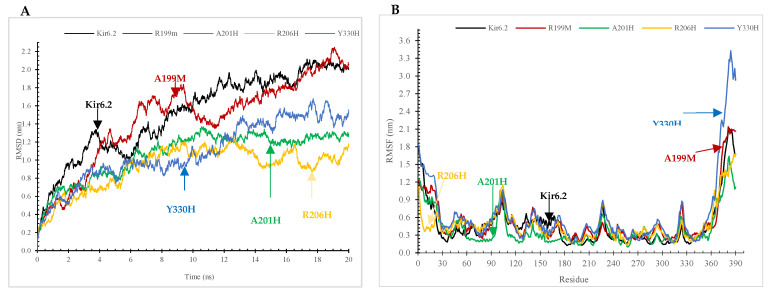
The root-mean-square deviation (RMSD) and root-mean-square fluctuation (RMSF) analyses conducted on both the wildtype and mutant variants of Kir6.2 protein. The RMSD (**A**) and RMSF (**B**) plots were created to analyze the backbone atoms of both the *KCNJ11* protein’s wildtype and mutant variants. These simulations were performed using GROMACS version 2023.3. The wildtype is denoted by the color black, whereas the mutants (M199R, R201H, R206H, and Y330H) are color-coded as red, green, yellow, and blue, respectively.

Prior to energy minimization, the protein structures were immersed within a balanced solvent environment along with ions. To ensure proper alignment of molecules within the structures, equilibration procedures using the NVT (canonical ensemble with temperature control) and NPT (canonical ensemble with pressure control) approaches were executed. These two phases, operating under the isothermal–isobaric ensemble, facilitated the maintenance of system temperature and pressure correspondingly. The computational tool “gms/grompp” was engaged to perform calculations for the NVT and NPT ensembles. The equilibration process involved manipulation of temperature, pressure, and density, resulting in the creation of a density plot through the NPT (constant number of particles, constant pressure, and constant temperature) ensemble. Figure 3 visually presents temperature profiles of protein systems in both native and mutant forms, recorded at 300 K. The narrative highlights discernible fluctuations in temperature across the conformations during the 100 ps equilibration phase. These observed temperature fluctuations were not entirely unexpected, as most conformations exhibited temperatures within favorable ranges. The native Kir6.2 demonstrates a temperature range with a minimum near 298 Kelvin (K) and a maximum approaching 304 K. The R199M mutation fluctuates between approximately 297 K and just above 303 K, showing the widest range of temperature variation among the mutations. The A201H mutation exhibits a more constrained range, with its lowest temperature around 298 K and the highest just below 303 K. The R206H mutation varies between nearly 297.5 K and 303 K, with a similar spread to R199M but with a slight upward shift in the temperature range. Lastly, the Y330H mutation maintains a temperature between almost 298 K and 303 K, with its fluctuations being slightly less than those of R199M and R206H (Supplementary Figure S1). The graph shows that, although all proteins undergo comparable temperature changes, there are unique patterns in the fluctuations for each mutant in comparison to the wild type, indicating that the mutations could affect the protein’s thermal stability or dynamics. The wild type Kir6.2 demonstrates a significantly more consistent temperature profile than the mutants, suggesting that the mutations may have influenced its inherent stability.

The wild type Kir6.2 protein displayed a pressure range of around 600 to 500 bar (Supplementary Figure S2) during a 100 ps MD simulation, indicating a dynamically stable yet flexible structure. The R199M mutation showed greater pressure fluctuations compared to the wild type, ranging from below −600 to above 600 bar, possibly indicating changes in dynamic stability or environmental interactions. The A201H and R206H mutations exhibited pressure fluctuations within a little more of a limited range than the wild type, fluctuating between around −500 and just above 500 bar, suggesting that these mutations may have a stabilizing effect on protein dynamics. The Y330H mutation exhibited pressure changes similar to the wild type, suggesting a minor impact on the protein’s structural stability. The findings emphasize how each mutation affects the pressure stability of the Kir6.2 protein differently, with each variant showing distinct profiles that may indicate variations in their functional activity or interaction with membrane lipids.

The density plot (Supplementary Figure S3) generated from the MD simulation illustrates the characteristics of the wild type Kir6.2 protein and its four mutations (R199M, A201H, R206H, Y330H) during a 100 ps period. The wild type Kir6.2 has exceptional stability, keeping a constant density of approximately 1000 kg/m^3^ with minimal variations. The R199M mutation shows slightly more variability, with density values fluctuating between around 1005 and 1015 kg/m^3^. The A201H and R206H mutations exhibit comparable patterns, both showing slight fluctuations around the density of 1010 kg/m^3^. The Y330H mutation has the highest density variation, ranging from around 1010 to 1025 kg/m^3^. The mutations have different effects on the protein’s density, with Y330H causing the most notable variation, possibly suggesting modifications in the protein’s volume or structural stability compared to the wild type Kir6.2.

#### 2.7.2. The Root-Mean-Square Deviation (RMSD) and Root-Mean-Square Fluctuation (RMSF) Analyses

By conducting a comparative investigation involving the RMSF and RMSD of wildtype Kir6.2 protein and its M199R, R201H, R206H, and Y330H mutations (Figure 3A,B), this study aims to unveil distinguishable dynamic behaviors. These findings provide insightful glimpses into the structural modifications induced by these mutations, which could potentially contribute to the molecular pathways underpinning the pathogenicity of DM. Employing an MD simulation approach, this study undertook a comparative analysis to elucidate the structural and functional attributes of the wildtype Kir6.2 protein and its four mutant variants. Figure 3A displays the RMSD of the Kir6.2 protein and four particular mutations (R199M, A201H, R206H, Y330H) throughout a 20 ns molecular dynamics simulation, offering a comparison of structural stability. The wild type Kir6.2 shows a gradual rise in RMSD, eventually stabilizing at approximately 1.5 angstroms, indicating some level of conformational flexibility prior to reaching equilibrium. The R199M variant displays a comparable early rise, but a higher peak around 2 angstroms, suggesting increased flexibility or divergence from the original structure. The A201H and R206H variants exhibit a slower increase, with A201H showing a distinct plateau at approximately 1.8 angstroms, indicating that these mutations might contribute some level of structural stiffness. The Y330H mutation shows a distinct behavior during the simulation, with its RMSD value increasing sharply towards the end, exceeding 3 angstroms. This suggests a notable destabilization or significant conformational change compared to the wild type and other mutations, potentially influencing the protein’s functional dynamics and interactions. The wild type Kir6.2 exhibits a steady RMSF value (Figure 3B) ranging from 0.1249 nm to 0.1249 nm, suggesting structural stability. The average RMSF is 0.4687 nm. The mutations exhibit enhanced flexibility: R199M has a maximum RMSF of 2.1412 nm and an average RMSF of 0.5161 nm, while A201H displays a minimum RMSF of 0.0993 nm, a maximum of 1.6393 nm, and an average RMSF of 0.3988 nm. The R206H mutation has a minimum RMSF of 0.1352 nm and a maximum of 1.6802 nm, with an average RMSF close to the wild type at 0.4735 nm. In contrast, the Y330H mutation displays the highest flexibility and dynamic change, with a maximum RMSF reaching 3.4299 nm and the highest average RMSF of 0.6363 nm, suggesting a significant effect on protein flexibility.

#### 2.7.3. Radius of Gyration (Rg) and Solvent-Accessible Surface Area (SASA)

The examination of the Rg and SASA for the wildtype and four mutations in Kir6.2 is imperative for comprehending their influence on protein structure and functions. These structural measures provide valuable insights into the compactness and exposure of amino acids, unveiling potential conformational alterations that contribute to the intricate interplay between these mutations in DM. 

**Figure 4:**
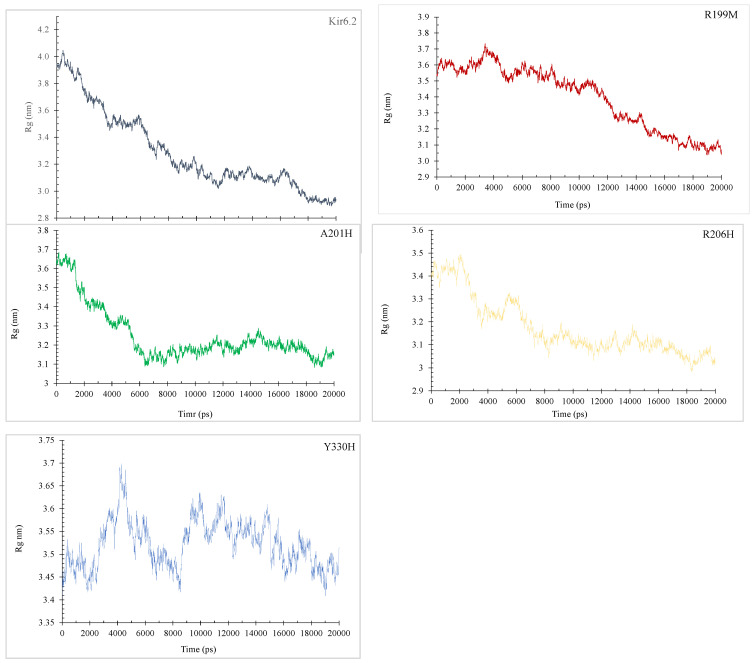
The radius of gyration (Rg) for Cα atoms in wildtype Kir6.2 and mutant forms, M199R, R201H, R206H, and Y330H.

The principles underlying the concepts of Rg and SASA hold significance across diverse fields of study. Rg pertains to the root-mean-square distance that all atoms, determined by their mass–weight, cover from their common center. This attribute facilitates a comprehensive grasp of the constituents of protein analysis. In Figure 4, a comparative assessment of normal and mutant protein structures at 300 K is presented through plotting the Rg values for alpha carbon atoms over time. 

The radius of gyration (Rg) plots for Kir6.2 and its variants (R199M, A201H, R206H, Y330H) over a 20,000 picosecond (ps) simulation offer insights on the compactness and stability of their structures (Figure 4). Kir6.2 initially has a greater radius of gyration (Rg), suggesting a less dense structure, which decreases from approximately 4.2 nm to 3.0 nm, indicating a notable compaction over time. The R199M mutation shows a decrease in initial compactness from around 3.8 nm to 3.0 nm, with a consistent tendency towards further compaction. The A201H protein starts at approximately 3.7 nm and stabilizes near 3.1 nm, indicating moderate stability with few changes in compactness. The R206H mutation initiates close to 3.6 nm and results in a progressive reduction in Rg to approximately 3.0 nm, suggesting a persistent tendency towards increased compactness. Y330H displays more pronounced fluctuations, with its Rg varying between 3.75 nm and 3.4 nm, indicating a consistent level of compactness but with heightened dynamism. The average radius of gyration (Rg) declines over time for all proteins. Mutations often begin with a lower Rg and maintain a lower average than the wild type. The Y330H variant shows the highest Rg values and potentially the least compact structure on average. 

**Figure 5:**
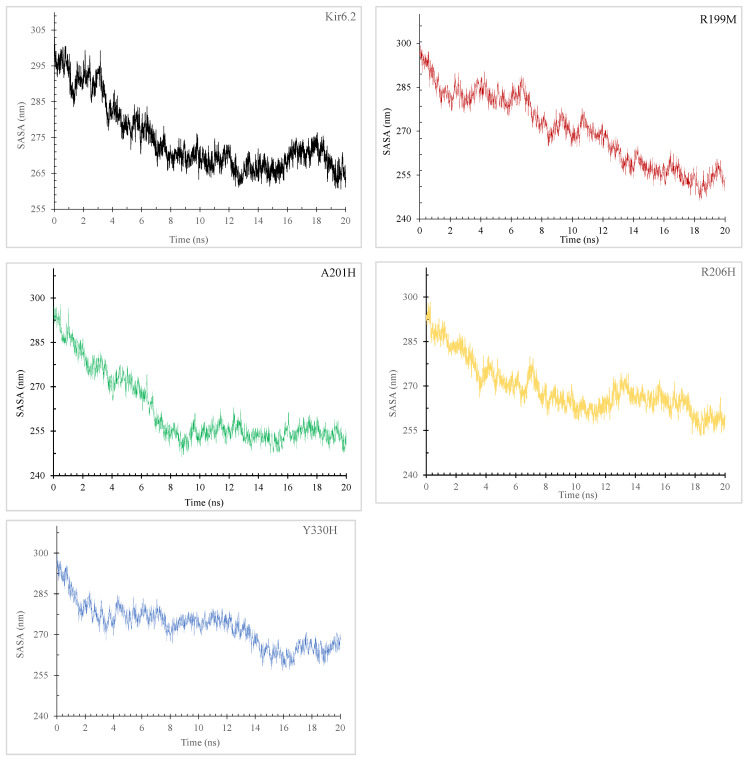
Analysis of solvent-accessible surface area (SASA) dynamics in Kir6.2 and its mutations M199R, R201H, R206H, and Y330H over a 20 ns molecular dynamics simulation.

An increase in SASA (Figure 5) value signifies structural expansion and surface area augmentation. The solvent-accessible surface area (SASA) graphs show the hydrodynamic characteristics and solvent exposure of the Kir6.2 protein and its four mutations (R199M, A201H, R206H, Y330H) during a 20ns simulation. The Kir6.2 protein exhibits a reduction in SASA from around 305 to 255 nm^2^ over the simulation, suggesting a shift towards a more compact and less exposed state. The R199M mutation exhibits a decrease in SASA, decreasing from just under 300 nm^2^ to stabilize around 255 nm^2^, indicating a conformational shift similar to the wild type. Both A201H and R206H initially start at around 300 nm^2^. However, A201H drops to about 250 nm^2^, whilst R206H exhibits greater variability and ends slightly higher. This indicates varying levels of residue exposure or compactness in their altered states. The Y330H mutation has the highest initial SASA of around 300 nm^2^, which decreases to 270 nm^2^. This suggests that, although this mutation follows the overall trend of reduced solvent exposure, it still maintains a higher level of exposure compared to other proteins, possibly due to a more open conformation or less compact folding. The fluctuations in SASA demonstrate the impact of mutations on the protein’s interaction with the surrounding solvent, which is crucial for its function and stability.

#### 2.7.4. Dynamic Hydrogen Bonding (HB) Changes in the Kir6.2 Protein Due to Genetic Variations

With the GROMACS software version 2023.3 and molecular dynamics simulations (Supplementary Figure S4), we looked closely at the patterns of HBs. Throughout a 20 ns MD simulation, the Kir6.2 protein and its variants R199M, A201H, R206H, and Y330H displayed unique HB patterns. The wild type Kir6.2 consistently maintained a stable number of HBs, fluctuating within a small range (Supplementary Figure S4). The R199M variant showed a similar HB profile to the wild type. The R201H variant exhibited a slightly decreased ability to form HBs. The R206H variant exhibited a comparable number of HBs to the wild type. The Y330H mutation showed significant variation in HB numbers (Supplementary Figure S4).

## 3. Discussion

Diabetes mellitus (DM) remains a big challenge to the healthcare system all over the world [29]. The prevalence of DM has increased, which necessitates methods for accurate diagnosis and treatment. Early detection and stratification of individual at risk to DM will help in delay or prevention of this disease because life style modifications such as weight loss, healthy diets, and regular exercise were reported to be sufficient for the prevention and delay of type 2 diabetes (T2D) [30]. The Kir6.2-G324R mutation alters the electrical activity of pancreatic beta cells and causes insufficient secretion of insulin and NDM [27]. In addition, the mutations that change the amino acid residues R201C, E23K, I337V, and S385C were reported in patients with NDM [26]. Moreover, gene variations in the *KCNJ11* gene are linked to an increased susceptibility to T2D [27,31]. The role of Kir6.2 mutations in the induction of insulin resistance and T2D is not clear. However, it has been suggested that the mutations that decrease ATP sensitivity may cause the susceptibility to T2D [27]. Furthermore, a mutation in the *KCNJ11* gene was reported to be associated with MODY [25,32,33]. For example, a *KCNJ11* gene variation resulting in an exchange of the conserved amino acid number 136 from arginine to cysteine (R136C) would lead to an alteration in the K_ATP_ channel’s structure, and may be associated with MODY [25]. In addition, the single nucleotide polymorphism, *KCNJ11* rs5219 (Lys23Gln), changes the charge of the ATP-binding region, reduces the channel’s sensitivity to ATP, and predisposes to T2D [8]. 

The Kir6.2 protein contains the binding regions of adenosine triphosphate (ATP) and phosphatidylinositol 4,5-diphosphate (PI(4,5)P2) [25]. This can cause inhibition (by ATP) or activation (by PI(4,5)P2 of the K_ATP_ channel) [25]. The K_ATP_ channels localized to pancreatic beta cells couple metabolism of energy and electrical activity, which have crucial roles in the secretion of insulin. When the glucose concentration is inadequate, the membrane potential of beta cells is influenced by the conductance of K_ATP_ channels and the cell membrane would be hyperpolarized or electrically silent [34]. When the concentration of glucose increases in the beta cells, it is metabolized into ATP [25,34]. This ATP binds the K_ATP_ channel [25]. Then, the K_ATP_ channel is closed, and the cell membrane is depolarized leading to electrical activity and open channels of the voltage-gated calcium ions. Then the calcium ions flow in and vesicles of insulin are secreted [25]. In the pancreatic beta cells, the activity of K_ATP_ channels may be influenced by mutations in the *KCNJ11* gene resulting in defective secretion of the insulin. The activating mutations could reduce the affinity of ATP to the beta cells’ K_ATP_ channels [25,34]. Then, the K_ATP_ channel would not close perfectly when stimulated by the increased glucose concentration leading to hyperpolarized cell membranes [25,34]. Therefore, calcium ions are not able to influx from the extracellular space and there will be impaired secretion of insulin resulting in the defective metabolism of glucose and, for example, NDM-impaired fasting sugar, glucose intolerance, and *KCNJ11*-MODY [25]. The Kir6.2-G324R mutation alters the electrical activity of the pancreatic beta cells, causing insufficient secretion of insulin and NDM [27]. 

In the current study, we employed bioinformatics tools to examine the nsSNPs that affect Kir6.2 stability, structure, and functions. Our results indicated that there are Kir6.2 variants (M199R, R201H, R206C, and Y330H) that potentially influence the stability, structure, and function of the Kir6.2 protein (Table 1, Table 2, Table 3 and Table 4), and hence these nsSNPs perhaps predispose to DM. 

Our results showed that Kir6.2 M199R affected the structure of the Kir6.2 protein (Figure 1, Figure 2, Figure 3, Figure 4 and Figure 5). Arginine (R) is a positively charged amino acid with a side chain containing a guanidinium group, whereas methionine (M) is a sulfur-containing hydrophobic amino acid [35]. This exchange may induce changes to the Kir6.2 protein structure and function. This result may be consistent with a study reporting that an exchange of hydrophobic amino acids to arginine affects the protein’s stability [36]. Furthermore, our MutPred analysis showed that the M199R mutation resulted in the loss of an allosteric site at R201, altered protein stability, and an altered disordered interface of Kir6.2 (Table 4), while our HOPE results showed that the M199R mutation resulted in altered size, charge, and hydrophobicity of Kir6.2, in addition to the disruption of Kir6.2 folding that may influence the Kir6.2 structure and function. 

Results indicated that the R206C variant influences the Kir6.2 structure (Figure 1, Figure 2, Figure 3, Figure 4 and Figure 5). In this mutation, the exchange from arginine to cysteine is a hydrophilic sulfur-containing amino acid [35]. According to our result, this mutation may cause a structural change in the Kir6.2 protein. This result may be in line with a study where the R206C mutation results in diseases [37]. Moreover, the HOPE result showed that R206C led to disrupted HBs, salt bridges, and domain interactions, indicating potential Kir6.2 functional effects. 

**Figure 6:**
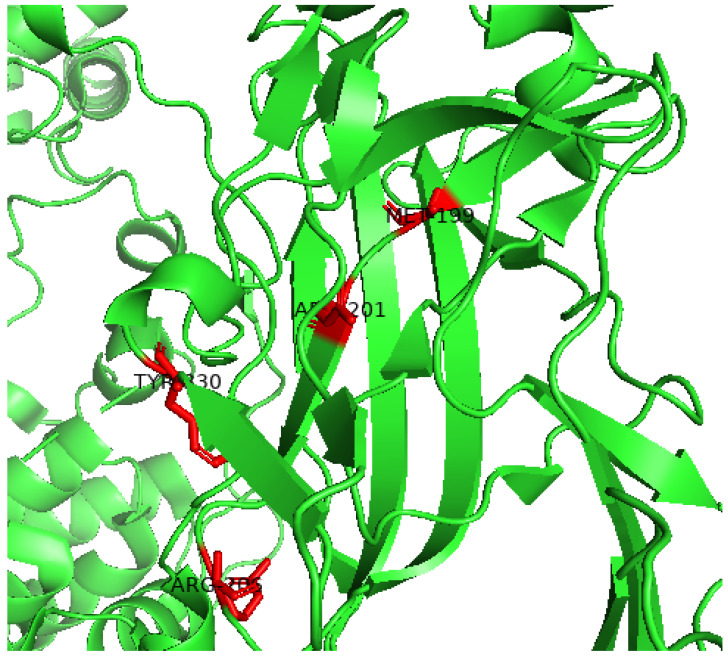
The 3D Kir6.2 protein with the site of the mutations indicated. The cartoon structure of the Kir6.2 protein (PDB 7TYT) with the sites of the M199R, R201H, R206H, and Y330H mutations indicated with red and stick structures. This figure was prepared using PyMol version 2.5.7. green: 3D of Kir6.2.

Results also showed that the R201H mutation could affect the Kir6.2 structure (Figure 1, Figure 2, Figure 3, Figure 4 and Figure 5). Arginine (R) 201 is changed to histidine (H), a unique amino acid as it can be neutral or positively charged at the physiological pH (Figure 6) [38]. The R201H mutation affects the protein binding properties to metal (Table 4). It may affect the binding of the Kir6.2 protein to the potassium that affects the K_ATP_ channel and induces DM. This result is consistent with a study reporting that the R201H mutation results in disease [39]. Moreover, the HOPE analysis indicated that the R201H results in Kir6.2 structural alteration, domain interactions, and potential effects on Kir6.2 function. 

Results indicated that the Y330H mutation influences the Kir6.2 structure (Figure 1, Figure 2, Figure 3, Figure 4 and Figure 5). In the Y330H mutation, the tyrosine, a hydrophilic amino acid, is substituted for histidine, which can be neutral or positively charged at the physiological pH (Figure 6) [38]. This can change the Kir6.2 structure and function [40]. The HOPE analysis revealed that the Y330H mutation might affect the Kir6.2 structure and function by disruption of HBs, multimer contacts as well as core structure alterations.

The Y330 mutation was reported to be associated with DM [28,41]. Tammaro et al., demonstrated that the Y330 mutation decreases ATP inhibition by hindering the ATP binding/transduction, and by the stabilization of the K_ATP_ channel’s intrinsic open state [28]. Pipatpolkai et al. reported that the major part of the binding cleft of the K_ATP_ channel is contributed by the Kir6.2 protein’s C-terminus, beta-sheet, and an alpha helix segments (residues tyrosine 330, phenylalanine 333, and glycine 334) [42]. The residues isoleucine 182 and tyrosine 330 are close to the ribose, and the adenine ring interacts with asparagine 48, arginine 50, and tyrosine 330 [42]. Changes in one of these amino residues (e.g., tyrosine 330) are associated with NDM [42,43,44], and a tyrosine 330 mutation decreases the ATP inhibition, and increases the open probability of the K_ATP_ channel [42]. Thus, the Y330H mutation leads to the Kir6.2 structural change, alters its sensitivity to ATP, and predisposes to NDM [28,42]. Our result indicated that the Y330H mutation affected the Kir6.2 structure (Figure 3, Figure 4 and Figure 5). This result is in line with the above studies, which reported the Y330 mutation’s effect on the Kir6.2 structure and function [28,42,43,44]. 

Our results from the study of the effects of temperature, pressure, and density on the Kir6.2 protein and mutant forms indicated that M199R, R201H, R206H, and Y330H might affect Kir6.2 stability, and therefore its structure and function may be affected (Supplementary Figures S1–S3). In addition, our RMSD and RMSF data results indicated that the Kir6.2 protein is more stable compared to the variants M199R, R201H, R206C, and Y330H, and that these variants influence the dynamics of the Kir6.2 protein (Figure 3). Furthermore, our radius of gyration (Rg) results (Figure 4) and SASA (Figure 5) showed that M199R, R201H, R206H, and Y330H affect folding, stability, compactness, and flexibility of Kir6.2 inside a biological environment [45]. Moreover, our results indicated that the pattern of HB of the wild type Kir6.2 is different from that of the mutant forms, R199M, R201H, R206C, and Y330H, indicating these variants affect the HB pattern of Kir6.2 (Supplementary Figure S4). The HBs are crucial for protein structure and function [46]. Results showed that during the 20 ns MD simulation, the Kir6.2 protein and its variants R199M, A201H, R206H, and Y330H displayed unique HB patterns, reflecting Kir6.2 stability and interaction dynamics. The wild type Kir6.2 consistently maintained a stable number of HBs, fluctuating within a small range, indicating steady intramolecular interactions. The R199M variant showed a similar HB profile to the wild type, indicating that this variant does not have a substantial impact on the protein’s capacity to create and uphold HBs. The R201H variant exhibited a slightly decreased ability to form HBs, which could potentially alter the protein’s local folding and interaction. The R206H variant exhibited a comparable number of HBs to the wild type, suggesting no interference with its HB network. The Y330H mutation showed a significant variation in HB numbers, indicating a dynamic and potentially less stable interaction network. The mutation Y330H could cause substantial conformational changes that affect the protein’s functionality and stability. The discovered patterns offer insights into how the mutations affect the dynamics of the Kir6.2 protein’s structure and function. This result is consistent with studies reporting that the mutations Y330C, Y330H, and Y330N are associated with diabetes [28,41]. It has been reported that substitution of the amino acid sequence of a protein may change the protein’s folding and stability, protein–protein interaction network, and function of the protein [47,48,49]. Genome-wide association studies reported the association of various diseases with specific loci [50,51,52,53]. The mutations affecting Kir6.2 function are implicated in DM [12,54]. We, therefore, hypothesize that the variants, M199R, R201H, R206H, and Y330H affect the stability of the Kir6.2 protein and predispose to DM. Future Kir6.2 functional studies (e.g., site-directed mutagenesis and protein–protein interaction [55,56,57]) and large-scale case control studies are recommended to verify these findings and broaden our understanding of *KCNJ11*-related pathogenesis. The limitations of the current study include that the MD simulation was for the monomeric Kir6.2 protein and not for the complete hetero-octameric K_ATP_ channel complex. The MD results are therefore preliminary. Future studies running MD simulations for models containing all K_ATP_ complex components, the Kir2.6 subunits, SUR1 subunits, membrane, and activator or inhibitor (e.g., PIP2 and ATP) are recommended.

## 4. Conclusions

In summary, our *in silico* analysis of Kir6.2 protein variants using machine-learning algorithms revealed potential deleterious nsSNPs potentially affecting the structure and function of Kir6.2 and causing diabetes mellitus (DM). Our molecular dynamic (MD) simulations indicated that these mutations (R199M, R201H, R206C, and Y330H) might influence the Kir6.2 protein’s stability and functional behavior. These findings offer insights into the molecular mechanisms for early identification of susceptible individuals. These MD simulation results are preliminary and future MD simulation analyses for Kir6.2 variants within a model system containing other components of the K_ATP_ channel (SUR1 subunits, membrane, and activators or inhibitors, e.g., PIP2 and ATP) are required to verify the effect of these mutations on the K_ATP_ channel. In addition, Kir6.2 protein variants experimental validations and large-scale case control studies are warranted to confirm these findings. 

## 5. Methodology

### 5.1. Access to the Database

The nucleotide sequence of *KCNJ11* (Accession Number: NM_000525) and the corresponding amino acid sequence of the encoded protein (Reference Sequence: NP_000516.2) were obtained from the National Centre for Biotechnology Information (NCBI) database. The data were accessed in FASTA format through the website http://www.ncbi.nlm.nih.gov (accessed on 8 October 2023). The SNP databases pertaining to the *KCNJ11* gene can be accessed through the SNP database hosted by the NCBI. The database can be accessed via the provided URL: https://www.ncbi.nlm.nih.gov/snp/?term=KCNJ11 (accessed on 8 October 2023). The data collected for analysis were sourced from the Online Mendelian Inheritance in Man (OMIM) database, focusing specifically on the *KCNJ11* gene and its associated protein. The information provided in this study was obtained from the OMIM database, which can be accessed at http://www.omim.org (accessed on 9 October 2023).

### 5.2. Anticipating the Impact of nsSNPs on the Functionality of the KNCJ11 Protein

The evaluation of potential impacts of nsSNPs was conducted using various online tools and servers, including SIFT [58] and PolyPhen2 [58,59], SNAP2 [60], and PANTHER [61]. These tools predict the potential impact of nsSNPs on protein function. The SIFT algorithm utilizes a tolerance index score for the assessment of SNPs, in which variations with scores lower than 0.05 are classified as deleterious. In contrast, the PolyPhen2 algorithm utilizes a numerical scoring system that spans from 0 to 1. A score of 0 signifies a neutral effect, while a score of 1 signifies the most pronounced negative effect. Another tool employed in this study is SNAP2, which enables the comparison of genomes and offers predictions concerning functional implications at the amino acid level. The SNAP2 algorithm employs a protein sequence and a collection of single amino acid variants (SAVs) as the input to predict the effect of each substitution on the molecular function of the protein. The range of prediction scores extends from −100, representing complete neutrality, to +100, indicating a substantial impact. The last tool, referred to as PANTHER, utilizes a scoring system ranging from −1 to 1 to evaluate the anticipated impact of the provided single nucleotide polymorphisms (SNPs) on protein functionality. The determination of the functionality of a single SNP can be achieved through the assessment of its score. A positive score signifies the functionality of the SNP and its impact on protein function, whereas a negative score implies the non-functionality of the SNP. Larger absolute values are suggestive of a more prominent anticipated influence on protein function. The *KCNJ11* gene’s SNPs, which were identified as prevalent within the fourth group of servers, were subsequently subjected to computational analysis.

### 5.3. Prediction of nsSNP Disease Associations on Kir6.2 Protein

The tools utilized for assessing the correlation between filtered SNPs and disease were predictor of human deleterious single nucleotide polymorphisms (PhD-SNP) and SNPS&GO [62,63,64,65]. These resources, accessible at http://snps.biofold.org/phd-snp/phd-snp.html (accessed on 15 October 2023) and http://snps-and-go.biocomp.unibo.it/snps-and-go/ (accessed on 15 October 2023), respectively, were employed in the investigation. The online bioinformatics tool known as PhD-SNP utilizes machine learning techniques to forecast the correlation between SNPs and various diseases. The scoring system assigns a numerical value ranging from 0 to 9 to SNPs based on their likelihood of causing a disease. This system categorizes SNPs as either disease-associated or neutral. The percentage of SNPs that have been accurately identified and validated in the context of a PhD study is 78% [60]. The SNPs&GO tool is a highly accurate resource for predicting disease-associated amino acid alterations at specific positions within a protein. It also provides functional classifications, achieving an impressive overall prediction accuracy rate of 82% [65]. The required data for SNPs & GO included the UniProt accession number (Q14654) of the Kir6.2 protein, as well as the specific positions of both the original and modified amino acids. 

### 5.4. Predicting Effects of nsSNPs on Kir6.2 Protein Stability

We used I-Mutant and MUpro [66] to find out how the most deleterious nsSNPs in the *KCNJ11* gene change the stability of the Kir6.2 protein. The I-Mutant tool, accessible at https://gpcr2.biocomp.unibo.it/cgi/predictors/I-Mutant3.0/I-Mutant3.0.cgi, (accessed on 16 October 2023) underwent training and testing using a dataset called ΔΔG Mut, which was sourced from ProTherm. The predictor has the capability to estimate the change in stability, as quantified by the ΔΔG value (kcal/mol), resulting from a single-site mutation. This estimation can be made using either a protein structure or a protein sequence as input. A ΔΔG value below zero signifies that the variant has a detrimental effect on protein stability. In contrast, a positive ΔΔG value signifies that the variant enhances the stability of the protein. The MUpro tool is a computational tool used for protein prediction, specifically focusing on the impact of single nucleotide polymorphisms (SNPs) on protein stability [66]. It utilizes a support vector machine (SVM) algorithm to predict whether a SNP will lead to an increase or decrease in protein stability. The tool provides a confidence score ranging from −1 to 1 to indicate the level of confidence in the prediction. To use the MUpro tool, you can access its website (http://www.ics.uci.edu/~baldig/mutation.html) (accessed on 17 October 2023) and input the amino acid sequence of the protein along with the specific position and type of the SNP. The tool will then analyze the sequence and provide predictions on the influence of the SNP on protein stability, along with a confidence score. Interpreting the results involves considering the confidence score, where a score closer to 1 suggests increased stability and a score closer to −1 suggests decreased stability. 

### 5.5. Prediction of Conserved Residues

In order to ascertain the evolutionary conservation of amino acids within a protein sequence, the ConSurf bioinformatics tool was employed [67]. This tool offers evolutionary profiles for individual amino acids within the protein, utilizing phylogenetic relationships among homologous sequences. The tool additionally provides predictions for the conservation score of each amino acid residue, which spans a range from 1 to 9. In this scoring system, residues with scores of 1 to 3 are considered variable, scores of 4 to 6 indicate medium conservation, and scores of 7 to 9 represent highly conserved residues. The aforementioned scores serve as indicators of the extent to which the amino acids exhibit evolutionary conservation. In order to utilize the application, it is necessary to input the protein sequence in FASTA format for the purpose of analysis.

### 5.6. MutPred

The computational tool known as MutPred is used for protein prediction, with a specific emphasis on evaluating the molecular and phenotypic consequences of amino acid variants [68]. This method employs a diverse range of molecular and structural mechanisms to forecast the impact of nsSNPs on the structure and stability of proteins. The software assigns hypothetical molecular changes and offers predictions regarding the potential effects of genetic variations on the structure and functionality of proteins. The MutPred tool can be accessed through its website at http://mutpred.mutdb.org/#qform (accessed on 17 October 2023). The input data for the tool consist of the protein’s amino acid sequence, as well as the specific position and type of the variant. Subsequently, the tool will conduct an analysis of the sequence and offer prognostications regarding the ramifications of the variant. This will encompass insights into alterations in solvent accessibility, pyrrolidone carboxylic acids, transmembrane regions, ubiquitylation, methylation, coiled coils, and helices. The interpretation of the findings necessitates a careful examination of the anticipated molecular modifications and their potential ramifications on the structure and functionality of proteins.

### 5.7. Hope

The HOPE server is an internet-based resource used for protein prediction, with a specific emphasis on evaluating the structural consequences arising from amino acid substitutions within a protein sequence [69]. The prediction of the impact of substitutions on protein structure is facilitated by the utilization of structural information obtained from diverse databases and software programs, including but not limited to WHAT IF web services, UniProt, and Reprof. Furthermore, the HOPE server provides estimations for disordered residues and the phi and psi dihedral angles of individual amino acids. The utilization of a deep neural network architecture is employed to augment the precision of its prognostications. The HOPE server can be accessed via its official website, which can be found at the following URL: https://www3.cmbi.umcn.nl/hope/, accessed on 18 October 2023. Upon accessing the website, the protein structure file is successfully uploaded. Subsequently, the server will undertake an analysis of the sequence and generate predictions regarding the potential structural consequences resulting from substitutions of amino acids.

### 5.8. PyoMol

PyMol Version 2.0 software was employed to map the sites of the exchanged amino acid residues. PyMol enables the examination of residues within the active site and serves as an open-source tool for molecular visualization. It excels in generating high-quality three-dimensional images of small molecules. As a result, we selected four potential mutants from the top seven nsSNPs predicted as deleterious mutants for subsequent molecular dynamics (MD) simulation analysis.

### 5.9. MD Simulations for Kir6.2 Protein and Its Variants

A molecular dynamics simulation lasting 20 nanoseconds was conducted using GROMACS-2023.1 with the 3D PDB ID: 7S5T [20]. The protein (monomeric Kir6.2) topology was prepared using the CHARMM36 force field, while the ligand topology was prepared using the General force field (CGenFF) server [70]. A dodecahedral unit cell form was utilized in the solvation process, along with periodic boundary conditions set at 10 Å to prevent atom interactions at the box border. Ions were incorporated through the steepest descent minimization approach, with sodium and chloride ions utilized for protein neutralization. Energy minimization was performed on the complex to prevent steric conflicts using the steepest descent minimization algorithm. The force cutoff was established at 10.0 kJ/mol, and a maximum of 50,000 steps were allowed. Two equilibration processes were conducted: NVT and NPT equilibration utilizing a modified Berendsen thermostat and leap-frog integrator for 50,000 steps, corresponding to 10 picoseconds. An MD simulation was conducted for 50 nanoseconds with a time step of 2 femtoseconds.

## Figures and Tables

**Table 1:** The table summarizes the predicted outcomes of amino acid substitutions in the *KCNJ11* gene based on SIFT (deleterious or tolerated), PolyPhen (damaging or benign), SNAP2, and PANTHER analyses.

			SIFT		Polyphen		SNAP2		PANTHER	
Variant ID	Alleles	nsSNP	Predication	Score	Score	Predication	Predication	Accuracy	Predication	Pdel
rs74339576	C>A	R301L	deleterious	0	1	probably damaging	Effect	95%	probably damaging	0.85
rs74339576	C>G	R301P	deleterious	0	1	probably damaging	Effect	95%	probably damaging	0.85
rs74339576	C>T	R301H	deleterious	0	1	probably damaging	Effect	95%	probably damaging	0.85
rs80356621	T>C	K170R	deleterious	0	0.997	probably damaging	Effect	95%	probably damaging	0.85
rs80356622	C>G	K170N	deleterious	0	1	probably damaging	Effect	95%	probably damaging	0.85
rs80356624	C>A	R201L	deleterious	0	1	probably damaging	Effect	95%	probably damaging	0.89
rs80356624	C>T	R201H	deleterious	0	1	probably damaging	Effect	95%	probably damaging	0.89
rs80356625	G>A	R201C	deleterious	0	1	probably damaging	Effect	95%	probably damaging	0.89
rs104894237	G>A	P254L	deleterious	0	1	probably damaging	Effect	95%	probably damaging	0.89
rs104894248	T>C	H259R	deleterious	0	0.998	probably damaging	Effect	95%	probably damaging	0.89
rs377091338	G>A	R301C	deleterious	0	1	probably damaging	Effect	85%	probably damaging	0.85
rs377091338	G>C	R301G	deleterious	0.01	1	probably damaging	Effect	95%	probably damaging	0.85
rs387906783	A>G	F60S	deleterious	0.2	1	probably damaging	Effect	85%	probably damaging	0.89
rs587783672	C>T	E227K	deleterious	0	1	probably damaging	Effect	85%	probably damaging	0.85
rs587783675	A>G	Y330H	deleterious	0.04	1	probably damaging	Effect	85%	probably damaging	0.85
rs750778014	C>A	R192L	deleterious	0	1	probably damaging	Effect	91%	probably damaging	0.85
rs761575495	G>A	T302I	deleterious	0.01	1	probably damaging	Effect	85%	probably damaging	0.57
rs761575495	G>T	T302N	deleterious	0.01	1	probably damaging	Effect	85%	probably damaging	0.57
rs775204908	G>A	R206C	deleterious	0	1	probably damaging	Effect	85%	probably damaging	0.85
rs797045637	C>A	G289V	deleterious	0.01	1	probably damaging	Effect	91%	probably damaging	0.89
rs1174593640	C>T	E292K	deleterious	0	0.997	probably damaging	Effect	91%	probably damaging	0.85
rs1404429785	C>T	G156R	deleterious	0	1	probably damaging	Effect	91%	probably damaging	0.89
rs1437510576	C>A	S265I	deleterious	0	1	probably damaging	Effect	91%	probably damaging	0.89
rs1554901747	C>A	R206L	deleterious	0	1	probably damaging	Effect	91%	probably damaging	0.85
rs2133379125	A>G	F315S	deleterious	0	1	probably damaging	Effect	85%	probably damaging	0.89
rs80356621	T>C	K170R	deleterious	0	0.997	probably damaging	Effect	95%	probably damaging	0.85
rs80356622	C>G	K170N	deleterious	0	1	probably damaging	Effect	95%	probably damaging	0.85
rs80356624	C>A	R201L	deleterious	0	1	probably damaging	Effect	95%	probably damaging	0.89
rs80356624	C>T	R201H	deleterious	0	1	probably damaging	Effect	95%	probably damaging	0.89
rs80356625	G>A	R201C	deleterious	0	1	probably damaging	Effect	95%	probably damaging	0.89
rs764444072	C>G	A87P	deleterious	0	0.998	probably damaging	Effect	85%	probably damaging	0.57
rs770553801	A>G	F60L	deleterious	0.02	1	probably damaging	Effect	85%	probably damaging	0.89
rs780511484	G>A	R192C	deleterious	0	1	probably damaging	Effect	85%	probably damaging	0.85
rs1435239409	T>C	K170E	deleterious	0	1	probably damaging	Effect	85%	probably damaging	0.85
rs1474444717	A>C	W68G	deleterious	0	1	probably damaging	Effect	91%	probably damaging	0.89
rs1591694925	T>G	Y304S	deleterious	0	1	probably damaging	Effect	95%	probably damaging	0.89
rs1591694946	T>G	T302P	deleterious	0.01	1	probably damaging	Effect	91%	probably damaging	0.57
rs1953581831	A>C	M199R	deleterious	0	0.997	probably damaging	Effect	85%	probably damaging	0.85
rs1953584995	C>T	G165D	deleterious	0	1	probably damaging	Effect	91%	probably damaging	0.89
rs1953585779	T>C	Q152R	deleterious	0	0.997	probably damaging	Effect	91%	probably damaging	0.85
rs1953586783	C>G	G134A	deleterious	0	0.998	probably damaging	Effect	91%	probably damaging	0.89

**Table 2:** Disease associations of nsSNPs in the Kir6.2 protein predicted by SNP&GO and PhD-SN algorithms.

			PhD-SNP		SNP&Go	
Variant ID	Alleles	nsSNP	Prediction	Score	Predication	Score
rs74339576	C>A	R301L	Disease	1	Disease	10
rs80356622	C>G	K170N	Disease	0	Disease	9
rs80356624	C>A	R201L	Disease	3	Disease	10
rs80356624	C>T	R201H	Disease	5	Disease	9
rs80356625	G>A	R201C	Disease	3	Disease	9
rs104894237	G>A	P254L	Disease	0	Disease	9
rs104894248	T>C	H259R	Disease	2	Disease	9
rs587783672	C>T	E227K	Disease	7	Disease	9
rs587783675	A>G	Y330H	Disease	4	Disease	9
rs775204908	G>A	R206C	Disease	1	Disease	9
rs797045637	C>A	G289V	Disease	2	Disease	9
rs1404429785	C>T	G156R	Disease	2	Disease	9
rs2133379125	A>G	F315S	Disease	4	Disease	9
rs80356622	C>G	K170N	Disease	0	Disease	9
rs80356624	C>A	R201L	Disease	3	Disease	10
rs80356624	C>T	R201H	Disease	5	Disease	9
rs80356625	G>A	R201C	Disease	3	Disease	9
rs764444072	C>G	A87P	Disease	3	Disease	8
rs780511484	G>A	R192C	Disease	3	Disease	9
rs1435239409	T>C	K170E	Disease	0	Disease	9
rs1591694946	T>G	T302P	Disease	2	Disease	9
rs1953581831	A>C	M199R	Disease	5	Disease	9
rs1953584995	C>T	G165D	Disease	4	Disease	9
rs1953585779	T>C	Q152R	Disease	5	Disease	9

**Table 3:** Projection of nsSNPs impact on Kir6.2 protein Stability (enhancing or diminishing).

Variant ID	Alleles	nsSNP	I-Mutant	MUpro
rs80356624	C>T	R201H	Decrease	Decrease
rs80356625	G>A	R201C	Decrease	Decrease
rs587783675	A>G	Y330H	Decrease	Decrease
rs775204908	G>A	R206C	Decrease	Decrease
rs2133379125	A>G	F315S	Decrease	Decrease
rs780511484	G>A	R192C	Decrease	Decrease
rs1953581831	A>C	M199R	Decrease	Decrease
rs1953585779	T>C	Q152R	Decrease	Decrease

**Table 4:** Assessing pathogenicity and associated functional changes of nsSNPs of Kir6.2 protein.

			MutPred		
Variant ID	Alleles	nsSNP	Effect	Score	Function Affected
rs80356624	C>T	R201H	-	0.914	Loss of allosteric site at R201; altered DNA binding; altered metal binding
rs80356625	G>A	R201C	-	0.948	Loss of allosteric site at R201; altered DNA binding
rs587783675	A>G	Y330H	-	0.868	Loss of acetylation at K332; loss of sulfation at Y330; altered metal binding; altered stability
rs775204908	G>A	R206C	-	0.949	Altered DNA binding
rs2133379125	A>G	F315S	-	0.946	Loss of allosteric site at F315; altered ordered interface
rs780511484	G>A	R192C	-	0.897	Loss of strand; altered metal binding
rs1953581831	A>C	M199R	-	0.957	Loss of allosteric site at R201; altered DNA binding; altered stability; altered disordered interface
rs1953585779	T>C	Q152R	no	0.903	No effect detected

(-): negative impact on protein function.

**Table 5:** Overview of nsSNPs of the Kir6.2 protein that have been identified.

Variant ID	Alleles	nsSNP	SIFT	PolyPhen	SNAP2	PANTHER	PhD-SNP	SNP&Go	I-Mutant	MUPro	ConSurf	MutPred
rs1953585779	T>C	Q152R	*	*	*	*	*	*	*	*	Cons, 9, BS	*
rs780511484	G>A	R192C	*	*	*	*	*	*	*	*	Cons, 8, EF	*
rs1953581831	A>C	M199R	*	*	*	*	*	*	*	*	Cons, 8, B	*
rs80356624	C>T	R201H	*	*	*	*	*	*	*	*	Cons, 9, BS	*
rs80356625	G>A	R201C	*	*	*	*	*	*	*	*	Cons, 9, BS	*
rs775204908	G>A	R206C	*	*	*	*	*	*	*	*	Cons, 9, EF	*
rs2133379125	A>G	F315S	*	*	*	*	*	*	*	*	Cons, 9, BS	*
rs587783675	A>G	Y330H	*	*	*	*	*	*	*	*	Cons, 7, B	*

* nsSNP effects Kir.6.2 protein.

## Data Availability

All data are available within the manuscript.

## Supplementary Materials

The following supporting information can be downloaded at: https://www.mdpi.com/article/10.3390/molecules29081904/s1.
