# Supplementary material for: Molecular Dynamics Simulation of Kir6.2 Variants Reveals Potential Association with Diabetes Mellitus
Source: Molecules. 2024 Apr 22;29(8):1904. doi: 10.3390/molecules29081904 (PMC11054064; doi:10.3390/molecules29081904)
Supplement: Supplementary file 1 [file molecules-29-01904-s001.zip › molecules-2929144-supplementary.pdf]

Supplementary Figure S1: Temperature fluctuations of Kir6.2 and variants

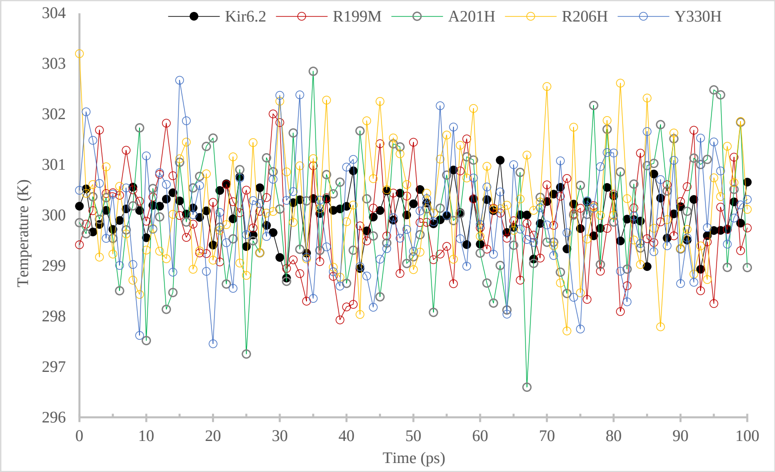

Supplementary Figure S1. The variations in temperature over time for both the wildtype and mutant forms of Kir6.2 protein. A chart depicting temporal temperature fluctuations of Kir6.2 protein and mutant forms, simulated through GROMACS software. The wildtype is denoted in black, whereas the mutants (M199R, R201H, R206H, and Y330H) are indicated in red, gray, yellow, and blue, respectively.

Supplementary Figure S2: Pressure fluctuation of Kir6.2 and variants.

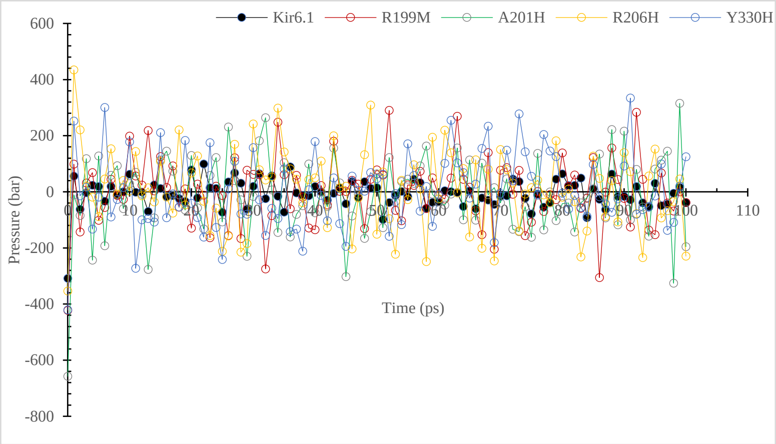

Supplementary Figure S2. The variations in pressure over time for both the wildtype and mutant forms of KCNJ11 proteins Kir6.2. A chart depicting temporal pressure fluctuations of Kir6.2 protein and mutant forms, simulated through GROMACS software. The wildtype is denoted in black, whereas the mutants (M199R, R201H, R206H, and Y330H) are indicated in red, green, yellow, and blue, respectively.

Supplementary Figure S3: Density fluctuations of Kir6.2 and variants.

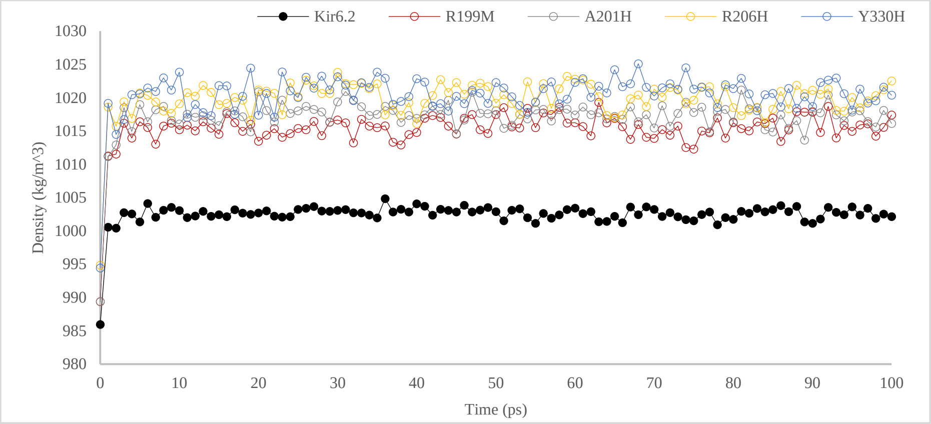

Supplementary Figure S3. The variations in density over time for both the wildtype and mutant forms of KCNJ11 protein Kir6.2: A chart depicting temporal density fluctuations of Kir6.2 wildtype and mutant forms, simulated through GROMACS software. The wildtype is denoted in black, whereas the mutants (M199R, R201H, R206H, and Y330H) are indicated in red, green, yellow, and blue, respectively. In contrast, the mutant variants exhibit higher-pressure values over the course of the simulation, measuring 1002.4, 1002.3, 1004.1, and 1004.5 Kg/m³ for the respective variants.

Supplementary Figure S4: Dynamic hydrogen bonding (HB)

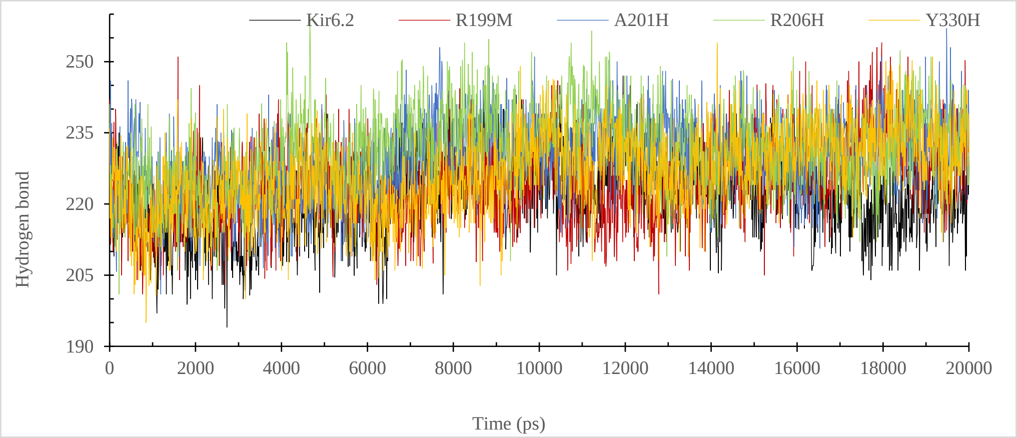

Supplementary Figure S4. Dynamic HB in Kir6.2 protein and mutant proteins. The HB fluctuations of Kir6.2 wildtype and mutant forms, simulated through GROMACS software. The wildtype is denoted in black, whereas the mutants (M199R, R201H, R206H, and Y330H) are indicated in red, blue, green, and yellow, respectively.
